# Whole-transcriptome gene expression profiling in an epidermolysis bullosa simplex Dowling-Meara model keratinocyte cell line uncovered novel, potential therapeutic targets and affected pathways

**DOI:** 10.1186/s13104-015-1783-7

**Published:** 2015-12-15

**Authors:** Julia Herzog, Raphaela Rid, Martin Wagner, Harald Hundsberger, Andreas Eger, Johann Bauer, Kamil Önder

**Affiliations:** Division of Molecular Dermatology, Department of Dermatology, Paracelsus Private Medical University Salzburg, Salzburg, Austria; Department of Medical and Pharmaceutical Biotechnology, University of Applied Sciences, Krems, Austria

**Keywords:** Epidermolysis bullosa simplex, Dowling-Meara, EBS-DM, Gene expression profiling

## Abstract

**Background:**

To be able to develop effective therapeutics for epidermolysis bullosa simplex (EBS), it is necessary to elucidate the molecular pathomechanisms that give rise to the disease’s characteristic severe skin-blistering phenotype.

**Results:**

Starting with a whole-transcriptome microarray analysis of an EBS Dowling-Meara model cell line (KEB7), we identified 207 genes showing differential expression relative to control keratinocytes. A complementary qRT-PCR study of 156 candidates confirmed 76.58 % of the selected genes to be significantly up-regulated or down-regulated (p-value <0.05) within biological replicates. Our hit list contains previously identified genes involved in epithelial cell proliferation, cell-substrate adhesion, and responses to diverse biological stimuli. In addition, we identified novel candidate genes and potential affected pathways not previously considered as relevant to EBS pathology.

**Conclusions:**

Our results broaden our understanding of the molecular processes dysregulated in EBS.

**Electronic supplementary material:**

The online version of this article (doi:10.1186/s13104-015-1783-7) contains supplementary material, which is available to authorized users.

## Background

**Fig. 1:**
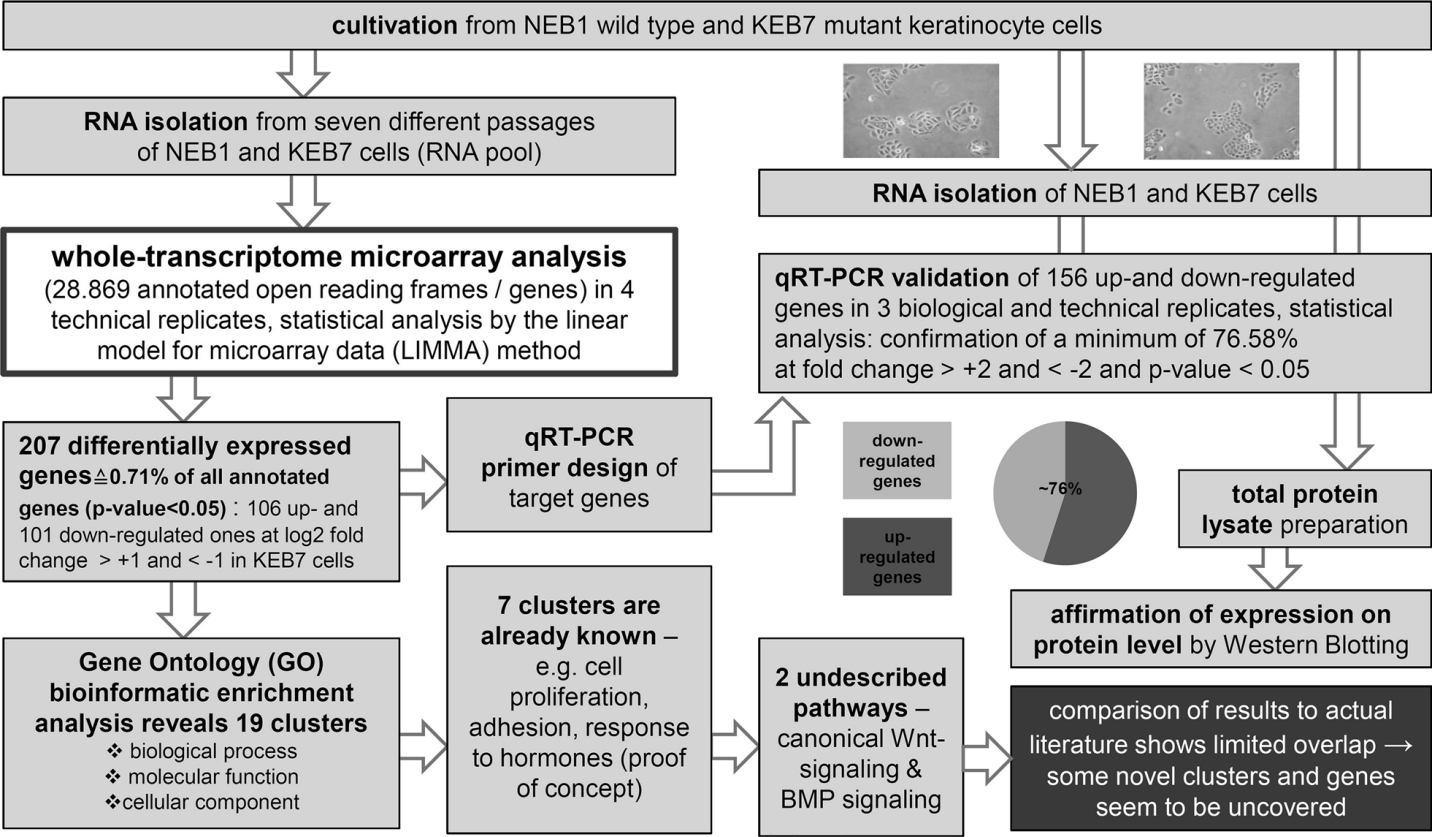
Experimental design of our study

KRT5 and KRT14 are the main stress-absorbing keratins in basal keratinocytes of the epidermis and related stratified epithelia. These rod-shaped proteins form heterodimeric units that interact to build up the cytoskeletal intermediate filament (IF) network, a resilient yet adaptable scaffold that maintains cellular structural stability and, in turn, normal skin integrity and function. Disruption of IF-organization as a consequence of keratin mutation is the basis of a number of inherited skin fragility syndromes [1, 2]. In the case of epidermolysis bullosa simplex (EBS), which exhibits several clinical variants [3], specific phenotypes can be largely correlated with the positions of missense mutations in structurally sensitive portions of either KRT5 or KRT14 [4]. Aberrant IF organization results in fragile epidermal basal cells that readily lyse following mild mechanical trauma or minor traction, leading to intraepithelial fluid accumulation and recurrent blister formation [5–8]. At the molecular level, this cytoskeletal collapse manifests as aggregates of misfolded keratins, along with activation of stress-response cascades [9, 10]. Detailed elucidation of the underlying pathomechanisms in EBS is an important prerequisite for developing innovative therapeutics; however, relatively few studies have focused on expression-profiling of mutant epidermal cells (summarized in Additional file 1: Table S1). In 2007, Lu et al. [11] reported the expression profile of KRT5^−/−^ EBS mouse epidermis, concentrating mainly on the regulation of inflammatory cytokines. Liovic et al. [12] investigated the response of the EBS keratinocyte cell lines KEB4 (mild EBS-loc phenotype, KRT14 mutation V270 M) and KEB7 (severe EBS Dowling-Meara (EBS-DM) phenotype, K14 mutation R125P) to hypo-osmotic stress in comparison to the wild-type cell line NEB1, and found dual-specificity phosphatases and their downstream targets ERK and p38 to be differentially regulated in EBS cells. A subsequent study by the same group identified differences in the expression profiles of cell-junction components in EBS versus wild-type cell lines [13]. More recent profiling studies reported aberrant expression of genes involved in keratinization, cell growth, proliferation, the immune response, and fatty acid metabolism in EBS [14]. Because there is limited overlap of the genes identified in those studies, further efforts seem necessary to fully elucidate EBS-relevant genes to better understand EBS pathology. We recently described the gene expression profile of KEB7 cells after applying suppression subtractive hybridization, and found dysregulated genes involved in keratinocyte differentiation, migration and wound healing [15]. Here, we follow-up that analysis with a more expansive expression profiling study, combining whole-transcriptome microarray examination with bioinformatics-assisted functional clustering, complementary qRT-PCR validation, and western blotting analysis of selected hits. Using this approach we were able to verify candidate genes previously described as being differentially expressed in EBS-DM, and to discover other differentially regulated genes not previously implicated in EBS pathology. A graphical summary of our experimental design is shown in Fig. 1.

## Methods

### Cell lines and culture conditions

The immortalized keratinocyte cell lines KEB7 (EBS-DM severe phenotype caused by the R125P mutation in KRT14) and NEB1 (wild-type control cell line) [16] were cultured in standard DMEM (HyClone Laboratories, GE Healthcare, South Logan, UT, USA) supplemented with 25 % Ham’s F12 nutrient mixture, 10 % fetal bovine serum (FBS), 1.8 × 10^−4^ M adenine, 0.4 µg/ml hydrocortisone, 5 µg/ml transferrin, 2 × 10^−11^ M liothyronine, 5 µg/ml insulin and 10 µg/ml EGF. Unless otherwise stated, all chemicals and reagents were obtained from Life Technologies™ (Karlsruhe, Germany) or Sigma-Aldrich Inc. (Taufkirchen, Germany). Cell lines were cultivated in standard 25 cm^2^ flasks (Techno Plastic Products AG, Trasadingen, Switzerland) at 37 °C under a 5 % CO_2_ atmosphere without fibroblast feeder cells.

### Total RNA isolation from cultivated KEB7 and NEB1 cells

Total RNA from cells grown to approximately 80 % confluence was isolated using an RNeasy Mini Kit (QiagenGmbH, Hilden, Germany) according to the manufacturer’s instructions. RNA was resuspended in nuclease-free water and quantified spectrophotometrically at 260 nm (DS-11 Spectrophotometer, DeNovix, Wilmington, DE, USA). RNA preparations were considered as suitable only if samples were of sufficient yield, exhibited intact bands on agarose gels, and displayed no spurious peaks or RNA degradation artifacts on the UV absorption spectrum. RNA samples were stored in aliquots at −80 °C.

### Whole-transcriptome microarray analysis

Total RNA, isolated from a pool of different passages of KEB7 versus NEB1 cells (p25 and p28–37), was used for probing a high-precision, whole-transcript Human Gene 1.0 ST array (Affymetrix, Santa Clara, CA, USA). The entire experimental pipeline, from sense target labeling to hybridization, washing, array scanning and final raw data capture, was performed at the Center of Excellence for Fluorescent Bioanalysis KFB, University of Regensburg, Germany, using standard Affymetrix protocols, reagents and instrumentation. Four microarrays per cell line were processed to account for technical and/or biological variability. The raw data were checked for quality, background-adjusted, quantile-normalized (by imposing the same distribution of gene signal intensities for each array used under the condition that the expression of most genes is relatively unaltered across mutant and wild-type cells), and statistically analyzed by the linear model for microarray data (LIMMA) method [17] using the Integromics Biomarker Discovery^®^ platform (IBD, Integromics, Granada, Spain). Assuming experimental deviations/errors of less than a log2-fold change of +1/−1, only candidates showing regulation greater than +2/−2 were subjected to further analysis. The respective statistical parameters were also used for the subsequent enrichment and qRT-PCR studies.

### Gene ontology enrichment and functional clustering of differentially expressed genes

Affymetrix IDs of differentially regulated genes were subjected to comprehensive bioinformatical analysis embedded within IBD. Gene ontology (GO) classification was performed to confirm enrichment of microarray-derived genes and to assign them to specific biological themes and functions. To ascertain GO-term enrichment of genes statistically overrepresented in the candidate hit list (log2-fold change of ≥ +1 and ≤ −1, adjusted p-value of <0.05), the software tool calculated a one-sided hypergeometric p-value (identical to the one-tailed version of Fisher’s exact test), so that terms with values below 0.05 can be considered to be significantly enriched.

### cDNA synthesis, primer design, and complementary qRT-PCR

Total RNA isolated from one passage of NEB1 and KEB7 cells (each cell line in technical triplicates) was treated with DNase I (Sigma-Aldrich, Taufkirchen, Germany) and then reverse transcribed into cDNA using an iScript™ cDNA Synthesis Kit (Bio-Rad Laboratories Inc., Hercules, CA USA) according to the manufacturer’s instructions. The resultant cDNAs served as template for complementary quantitative real-time PCR (qRT-PCR) in a 96-well plate format using GoTaq^®^qPCR Master Mix (Promega Corporation, Madison, WI, USA) and a CFX96™ instrument (Bio-Rad Laboratories). Using Batch Entrez (http://www.ncbi.nlm.nih.gov/sites/batchentrez) and WIBR UTR extractor (http://jura.wi.mit.edu/bioc/tools/utrs/), we extracted the coding sequences (highlighted within the downloaded mRNAs) of the 79 most promising up-regulated and the 79 most promising down-regulated genes uncovered in the microarray analysis to automatically design oligonucleotides with similar properties (uniform GC content, comparable melting temperature, optimal length and similar product size; BATCH Primer 3 program, http://probes.pw.usda.gov/cgi-bin/batchprimer3/batchprimer3.cgi). Forward and reverse primer stocks (100 pmol/µl, see Additional file 2: Table S2) were purchased in 96-well format from Life Technologies GmbH (Karlsruhe, Germany). Differential expression of selected candidate genes was investigated by performing three independent PCR-runs (biological and technical replicates) in an in-house developed and established 96-well qRT-PCR array format. Seven constitutively expressed housekeeping genes (ACTB, ANXA1, B2 M, GAPDH, HPRT1, RPL13A, and TUBB) served as internal controls for data normalization as well as determination of experimental variance. Fold inductions and statistical significance were assessed via RealTimeStatMiner^®^ from Integromics^®^ that calculates the relative quantity (RQ) and log2-fold change using ΔΔCt (cycle threshold) and efficiency correction [18]. A parametric LIMMA moderate t-test [17] and a Benjamini–Hochberg false discovery rate (FDR) p-value correction [19], included as standard default parameters of the software (log2-fold change of +1/−1, p-value <0.05), were applied in the data analysis. The meaning of an FDR-adjusted p-value is that, if all genes with a p-value below a threshold of 0.05 are picked as differentially expressed, then the expected proportion of false discoveries in the selected group is controlled to be less than the threshold value, in this case 5 %.

### KEB7/NEB1 total protein lysates and Western blotting

For preparation of KEB7 versus NEB1 protein lysates (using three 75-cm^2^ flasks per cell line), the culture medium was removed, the cells were rinsed twice with sterile PBS, and then lysed in RIPA buffer (Radio-Immunoprecipitation Assay buffer; 150 mM NaCl, 1 % IGEPAL^®^ CA-630, 0.5 % deoxycholate, 0.1 % SDS, 50 mM Tris–HCl, pH 8.0, Sigma-Aldrich) supplemented with 1× Protease Inhibitor Cocktail (GE Healthcare, USA) for 10 min at RT (room temperature) and a further 20 min at −80 °C. Cells were detached with a cell scraper, and the resultant total protein lysates transferred to 1.5-ml tubes and centrifuged at maximum speed for 15 s to remove cellular debris. Protein in the supernatants was photometrically quantified by a standard Bradford assay (Bio-Rad, Hercules, CA, USA), and the samples were frozen at −80 °C for long-term storage. 10 µg of protein lysate per slot were subjected to SDS-PAGE (sodium dodecyl sulfate polyacrylamide gel electrophoresis) and then transferred onto Amersham™ Hybond™ ECL nitrocellulose membranes (GE Healthcare). After blocking of the membrane in 3 % nonfat milk in Tris-buffered saline (TBS, pH 8.0, Sigma-Aldrich) containing 0.05 % Tween-20 for 1 h at RT, target-specific primary antibodies diluted 1:1000 in TBS with 0.05 % Tween-20 were added overnight at 4 °C with gentle agitation (Wnt-5a antibody, sc-365370, mouse monoclonal, Santa Cruz Biotechnology, Inc.; Cytokeratin 19 antibody, 11-120-C020, mouse monoclonal, EXBIO Praha a.s.; SOX2 antibody, D1951-1NB-1-1R1M1M3/1C16_110120, mouse monoclonal, ABMART; GAPDH antibody, TA302944-100, goat polyclonal, OriGene; antibodies were pre-tested for adequate performance in different human cell lysates; data not shown). The next day the membranes were incubated in horseradish-peroxidase (HRP)-labeled secondary antibodies (1:10,000, polyclonal rabbit anti-mouse IgG/HRP, polyclonal rabbit anti-goat IgG/HRP, Dako Denmark A/S, Glostrup, Denmark) for 1 h at RT. Blots were developed using Immobilon™ Western Chemiluminescent HRP Substrate (Merck/Millipore, Darmstadt, Germany) and signals detected with a ChemiDoc XRS system (Bio-Rad Laboratories). Band intensities were densitometrically quantified by using the image-processing program ImageJ (http://rsb.info.nih.gov/ij/).

## Results

### Global transcriptional profiling uncovers 207 differentially expressed genes in the EBS-DM cell line KEB7

Transcriptional profiling using the Affymetrix Human Gene 1.0 ST gene chip platform offers coverage of nearly the entire transcriptome, defined by 28,869 annotated open reading frames/genes. We applied this platform to study the expression pattern of genes influenced by the cytoskeletal collapse caused by the R125P mutation in KRT14 in KEB7 cells. Table 1 summarizes the results of our microarray profiling (four technical replicates each) using a pool of different passages (biological replicates) of KEB7 cells relative to NEB1 wild-type control cells. A LIMMA statistical test comparing mutant versus wild-type cells identified 106 up-regulated and 101 down-regulated genes with significantly altered expression levels (log2-fold change ≥+1 and ≤−1, adjusted p-value <0.05) out of 382 differentially expressed genes (failed at log2-fold change and p-value set).

**Table 1:** Genes regulated in the EBS-DM model keratinocyte cell line KEB7 identified by microarray analysis

Microarray candidate genes (accession number)	Regulation microarray	log2 fold-change microarray	Fold-change qRT-PCR	Significance of qRT-PCR (fold-change set −2)
KRT19 (NM_002276)	Significant down-regulated	−5.74	−1633.0	Significant
MIR492 (NR_030171)	Significant down-regulated	−4.46	n.d.	n.d.
KYNU (NM_003937)	Significant down-regulated	−4.20	−276.6	Significant
H19, MIR675 (NR_030533)	Significant down-regulated	−4.12	n.d.	n.d.
EYA4 (NM_004100)	Significant down-regulated	−4.03	n.d.	n.d.
PDZK1 (NM_002614)	Significant down-regulated	−3.86	−8.0	Significant
TMPRSS15 (NM_002772)	Significant down-regulated	−3.85	n.d.	n.d.
OLFM4 (NM_006418)	Significant down-regulated	−3.73	−8.4	Significant
SLC38A4 (NM_018018)	Significant down-regulated	−3.67	−12.3	Significant
BST2 (NM_004335)	Significant down-regulated	−3.53	−137.3	Significant
ITGBL1 (NM_004791)	Significant down-regulated	−2.99	n.d.	n.d.
EDIL3 (NM_005711)	Significant down-regulated	−2.91	n.d.	n.d.
PPARGC1A (NM_013261)	Significant down-regulated	−2.79	−964.3	Significant
GALNT5 (NM_014568)	Significant down-regulated	−2.73	−78.3	Significant
CDR1 (NM_004065)	Significant down-regulated	−2.71	n.d.	n.d.
FKBP10 (NM_021939)	Significant down-regulated	−2.63	−26.8	Significant
GIPC2 (NM_017655)	Significant down-regulated	−2.55	−71.4	Significant
AMOT (NM_001113490)	Significant down-regulated	−2.53	−3015.8	Significant
ZNF114 (NM_153608)	Significant down-regulated	−2.52	−10.2	Significant
CLEC2B (NM_005127)	Significant down-regulated	−2.50	−182.1	Significant
FAM198B (NM_001031700)	Significant down-regulated	−2.50	−14.0	Significant
SLC2A3 (NM_006931)	Significant down-regulated	−2.48	−88.7	Significant
CAPNS2 (NM_032330)	Significant down-regulated	−2.43	−9.0	Significant
TBX18 (NM_001080508)	Significant down-regulated	−2.43	−89.4	Significant
LRCH2 (NM_020871)	Significant down-regulated	−2.36	−44.2	Significant
NEFM (NM_005382)	Significant down-regulated	−2.34	−7.7	Significant
CPT1C (NM_001136052)	Significant down-regulated	−2.32	−13.4	Significant
ZNF43 (NM_001256648)	Significant down-regulated	−2.22	−9.7	Significant
LY75, LY75-CD302 (NM_002349)	Significant down-regulated	−2.17	−8.6	Significant
LOC100129717, NEFL (NM_006158)	Significant down-regulated	−2.16	n.d.	n.d.
GLDC (NM_000170)	Significant down-regulated	−2.14	−307.6	Significant
TMTC1 (NM_001193451)	Significant down-regulated	−2.13	−6.7	Significant
SLCO1B3 (NM_019844)	Significant down-regulated	−2.10	−988.0	Significant
SMOC2 (NM_022138)	Significant down-regulated	−2.09	n.d.	n.d.
SLC6A14 (NM_007231)	Significant down-regulated	−2.02	−4.9	Significant
SLC24A3 (NM_020689)	Significant down-regulated	−1.97	−53.0	Significant
EPSTI1 (NM_001002264)	Significant down-regulated	−1.96	−10.8	Significant
SATB2 (NM_001172509)	Significant down-regulated	−1.94	−224.2	Significant
HSD17B2 (NM_002153)	Significant down-regulated	−1.89	−59.8	Significant
GHR (NM_000163)	Significant down-regulated	−1.87	n.d.	n.d.
TFPI2 (NM_006528)	Significant down-regulated	−1.86	n.d.	n.d.
AKR1B10 (NM_020299)	Significant down-regulated	−1.86	−3.8	Significant
ARHGAP28 (NM_001010000)	Significant down-regulated	−1.81	n.d.	n.d.
LOC100506941, NNMT (NM_006169)	Significant down-regulated	−1.81	n.d.	n.d.
GPC3 (NM_001164617)	Significant down-regulated	−1.81	−427.1	Significant
IFITM3 (JQ610621)	Significant down-regulated	−1.80	−8.7	Significant
HOXD10 (NM_002148)	Significant down-regulated	−1.79	−100.8	Significant
MSX2 (NM_002449)	Significant down-regulated	−1.77	−9.5	Significant
IL17RB (NM_018725)	Significant down-regulated	−1.75	−9.1	Significant
BLMH (NM_000386)	Significant down-regulated	−1.74	−4.8	Significant
SOX2 (NM_003106)	Significant down-regulated	−1.72	n.d.	n.d.
HIST1H	Significant down-regulated	−1.70	n.d.	n.d.
SLC9A2 (NM_003048)	Significant down-regulated	−1.69	−17.0	Significant
CPNE1, RBM12 (NM_006047)	Significant down-regulated	−1.68	−14.9	Significant
WDR17 (NM_170710)	Significant down-regulated	−1.60	−11.2	Significant
RB1 (NM_000321)	Significant down-regulated	−1.58	−4.4	Significant
DPYD (NM_000110)	Significant down-regulated	−1.57	−3.7	Significant
PCCA (NM_000282)	Significant down-regulated	−1.56	n.d.	n.d.
ZNF570 (NM_001300993)	Significant down-regulated	−1.51	n.d.	n.d.
PRTFDC1 (NM_020200)	Significant down-regulated	−1.49	n.d.	n.d.
GLRX (NM_002064)	Significant down-regulated	−1.47	−2.8	Significant
SLC7A2 (NM_003046)	Significant down-regulated	−1.45	−1.5	Non-significant
PPP1R16B (NM_015568)	Significant down-regulated	−1.44	−8.0	Significant
IKZF3 (NM_012481)	Significant down-regulated	−1.41	−1.8	Non-significant
GTF2H2D	Significant down-regulated	−1.40	−4.2	Significant
CDK14 (NM_001287135)	Significant down-regulated	−1.40	n.d.	n.d.
REPS2 (NM_004726)	Significant down-regulated	−1.38	−4.5	Significant
MEST (NM_002402)	Significant down-regulated	−1.38	n.d.	n.d.
CYP4V2 (NM_207352)	Significant down-regulated	−1.35	−4.7	Significant
GPR143 (NM_000273)	Significant down-regulated	−1.35	−2.1	Significant
GTF2H2 (NM_001515)	Significant down-regulated	−1.35	−4.0	Significant
CYP7B1 (NM_004820)	Significant down-regulated	−1.32	n.d.	n.d.
BCL11A (NM_022893)	Significant down-regulated	−1.31	−3.6	Significant
MERTK (NM_006343)	Significant down-regulated	−1.30	−5.0	Significant
PRDM5 (NM_018699)	Significant down-regulated	−1.28	−6.1	Significant
ACOXL (NM_001142807)	Significant down-regulated	−1.28	−11.6	Significant
AHCY (NM_000687)	Significant down-regulated	−1.27	−2.0	Significant
ARMCX2 (NM_177949)	Significant down-regulated	−1.24	−22.5	Significant
PAX6 (NM_000280)	Significant down-regulated	−1.23	−14.2	Significant
HOXD11 (NM_021192)	Significant down-regulated	−1.23	−49.4	Significant
SMARCA1 (NM_003069)	Significant down-regulated	−1.22	−4.1	Significant
IFI44L (NM_006820)	Significant down-regulated	−1.22	−57.0	Significant
GALNTL4	Significant down-regulated	−1.20	n.d.	n.d.
PITRM1 (NM_001242307)	Significant down-regulated	−1.19	−2.3	Significant
CRIP2 (NM_001312)	Significant down-regulated	−1.18	n.d.	n.d.
NAP1L5 (NM_153757)	Significant down-regulated	−1.15	−20.7	Significant
IPO7 (NM_006391)	Significant down-regulated	−1.15	n.d.	n.d.
SAAL1 (NM_138421)	Significant down-regulated	−1.13	n.d.	n.d.
KRTCAP3 (NM_001168364)	Significant down-regulated	−1.12	n.d.	n.d.
FAM159A (NM_001042693)	Significant down-regulated	−1.11	n.d.	n.d.
EYA1 (NM_172060)	Significant down-regulated	−1.09	n.d.	n.d.
PIGU (NM_080476)	Significant down-regulated	−1.09	−3.2	Significant
CDC25B (NM_021873)	Significant down-regulated	−1.09	n.d.	n.d.
NKX2-6 (NM_001136271)	Significant down-regulated	−1.07	n.d.	n.d.
HTATIP2 (NM_001098520)	Significant down-regulated	−1.05	n.d.	n.d.
ILK (NM_004517)	Significant down-regulated	−1.05	n.d.	n.d.
ACSF2 (NM_001288968)	Significant down-regulated	−1.04	n.d.	n.d.
PDZD2 (NM_178140)	Significant down-regulated	−1.03	n.d.	n.d.
CENPH (NM_022909)	Significant down-regulated	−1.03	n.d.	n.d.
TOX (NM_014729)	Significant down-regulated	−1.02	n.d.	n.d.
VSTM2L (NM_080607)	Significant down-regulated	−1.01	n.d.	n.d.
SYT17 (NM_016524)	Significant down-regulated	−1.01	n.d.	n.d.

Microarray candidate genes (accession number)	Regulation microarray	log2 fold-change microarray	Fold-change qRT-PCR	Significance of qRT-PCR (fold-change set +2)
TDRD12 (NM_001110822)	Significant up-regulated	3.98	618.1	Significant
NEFH (NM_021076)	Significant up-regulated	3.45	1174.9	Significant
NLRP2 (NM_017852)	Significant up-regulated	3.39	154.3	Significant
TSPYL5 (NM_033512)	Significant up-regulated	3.20	n.d.	n.d.
KLK5 (NM_012427)	Significant up-regulated	3.18	62.8	Significant
ENPP1 (NM_006208)	Significant up-regulated	3.09	6.1	Significant
GJB6 (NM_001110219)	Significant up-regulated	2.98	24.4	Significant
ZFP42 (NM_174900)	Significant up-regulated	2.84	313.7	Significant
RNF212 (NM_001131034)	Significant up-regulated	2.59	n.d.	n.d.
DDX43 (NM_018665)	Significant up-regulated	2.51	n.d.	n.d.
DKK1 (NM_012242)	Significant up-regulated	2.40	45.3	Significant
CYYR1 (NM_052954)	Significant up-regulated	2.36	19.6	Significant
C10orf99 (NM_207373)	Significant up-regulated	2.33	10.6	Significant
SYCP2 (NM_014258)	Significant up-regulated	2.23	10.2	Significant
ZNF136 (NM_003437)	Significant up-regulated	2.17	n.d.	n.d.
CCDC144A (NM_014695)	Significant up-regulated	2.11	n.d.	n.d.
PRICKLE1 (NM_153026)	Significant up-regulated	2.08	52.4	Significant
SLC44A5 (NM_152697)	Significant up-regulated	2.06	100.5	Significant
PLA2G7 (NM_005084)	Significant up-regulated	2.05	18.6	Significant
CCDC144A, CCDC144B, CCDC144C	Significant up-regulated	2.01	n.d.	n.d.
MOXD1 (NM_015529)	Significant up-regulated	1.97	15.9	Significant
ZNF700 (NM_144566)	Significant up-regulated	1.96	n.d.	n.d.
WNT5A (NM_003392)	Significant up-regulated	1.94	6.9	Significant
WISP3 (CCN6) (NM_003880)	Significant up-regulated	1.92	103.8	Significant
ARHGEF9 (NM_015185)	Significant up-regulated	1.88	25.0	Significant
HSD17B11 (NM_016245)	Significant up-regulated	1.87	3.1	Significant
BGN (NM_001711)	Significant up-regulated	1.87	1.8	Non-significant
ADAMTSL3 (NM_207517)	Significant up-regulated	1.84	26.4	Significant
FAM102B (NM_001010883)	Significant up-regulated	1.78	2.5	Significant
SGMS1 (NM_147156)	Significant up-regulated	1.76	2.9	Significant
ARHGAP29 (NM_004815)	Significant up-regulated	1.75	4.1	Significant
H2AFY2 (NM_018649)	Significant up-regulated	1.74	1.8	Non-significant
SLC15A2 (NM_021082)	Significant up-regulated	1.73	4.6	Significant
ROBO1 (NM_002941)	Significant up-regulated	1.73	5.6	Significant
ERCC6, PGBD3 (NM_000124)	Significant up-regulated	1.68	6.0	Significant
NREP (NM_004772)	Significant up-regulated	1.67	3.0	Significant
KIAA1324L (NM_001142749)	Significant up-regulated	1.62	149.8	Significant
SNORD116-21 (NR_003335)	Significant up-regulated	1.62	n.d.	n.d.
HOXA9 (NM_152739)	Significant up-regulated	1.59	1.3	Non-significant
ROR1 (NM_005012)	Significant up-regulated	1.59	9.5	Significant
RPS23 (NM_001025)	Significant up-regulated	1.58	n.d.	n.d.
MAN1A1 (NM_005907)	Significant up-regulated	1.55	1.0	Non-significant
STOX1 (NM_152709)	Significant up-regulated	1.54	−1.1	Non-significant
RPL10, SNORA70 (NM_006013)	Significant up-regulated	1.53	−1.1	Non-significant
CCDC144C	Significant up-regulated	1.52	n.d.	n.d.
ZFAND4 (NM_174890)	Significant up-regulated	1.50	2.5	Significant
NOX5, SPESP1 (NM_024505)	Significant up-regulated	1.50	n.d.	n.d.
EVC2 (NM_147127)	Significant up-regulated	1.46	n.d.	n.d.
SELENBP1 (NM_003944)	Significant up-regulated	1.45	64.8	Significant
ZNF334 (NM_018102)	Significant up-regulated	1.43	15.6	Significant
PTPN20C (NM_001042357)	Significant up-regulated	1.43	n.d.	n.d.
IRX4 (NM_001278632)	Significant up-regulated	1.41	90.4	Significant
PTPN20A (NM_001042357)	Significant up-regulated	1.41	29.5	Significant
PTPN20B (NM_001042357)	Significant up-regulated	1.40	n.d.	n.d.
PNMAL1 (NM_018215)	Significant up-regulated	1.40	2.9	Significant
NID1 (NM_002508)	Significant up-regulated	1.38	1.8	Non-significant
ZNF32 (NM_006973)	Significant up-regulated	1.37	3.5	Significant
ASAH2 (NM_019893)	Significant up-regulated	1.37	n.d.	n.d.
FLJ20444, LOC554249	Significant up-regulated	1.36	n.d.	n.d.
TP53INP1 (NM_033285)	Significant up-regulated	1.35	1.6	Non-significant
SLC16A4 (NM_004696)	Significant up-regulated	1.34	16.4	Significant
FRG1B (NR_003579)	Significant up-regulated	1.32	n.d.	n.d.
AHI1 (NM_001134831)	Significant up-regulated	1.32	3.7	Significant
MSLN (NM_005823)	Significant up-regulated	1.30	n.d.	n.d.
ELAVL2 (NM_004432)	Significant up-regulated	1.29	4.3	Significant
SLC16A9 (NM_194298)	Significant up-regulated	1.28	2.4	Significant
FAM25A, FAM25B, FAM25C, FAM25G (NM_001146157)	Significant up-regulated	1.28	2.5	Significant
MAPK8 (NM_002750)	Significant up-regulated	1.28	2.2	Significant
CDC14B (NM_003671)	Significant up-regulated	1.26	3.0	Significant
GABPB2 (NM_144618)	Significant up-regulated	1.26	2.3	Significant
DENND1B (NM_144977)	Significant up-regulated	1.25	n.d.	n.d.
TCHH (NM_007113)	Significant up-regulated	1.23	28.0	Significant
LMF1 (NM_022773)	Significant up-regulated	1.22	5.1	Significant
CSTF2T (NM_015235)	Significant up-regulated	1.22	2.2	Significant
IFFO2 (NM_001136265)	Significant up-regulated	1.21	n.d.	n.d.
SGK1 (NM_005627)	Significant up-regulated	1.19	4.2	Significant
HOXC10 (NM_017409)	Significant up-regulated	1.19	2.2	Significant
STRBP (NM_018387)	Significant up-regulated	1.18	n.d.	n.d.
UAP1 (NM_003115)	Significant up-regulated	1.18	2.3	Significant
POPDC2 (NM_022135)	Significant up-regulated	1.18	3.9	Significant
DSEL (NM_032160)	Significant up-regulated	1.17	−1.2	Non-significant
AOX1 (NM_001159)	Significant up-regulated	1.16	1.4	Non-significant
ZNF502 (NM_033210)	Significant up-regulated	1.16	500.9	Significant
LRP12 (NM_013437)	Significant up-regulated	1.15	n.d.	n.d.
ADHFE1 (NM_144650)	Significant up-regulated	1.12	1.8	Non-significant
FAM21D	Significant up-regulated	1.11	n.d.	n.d.
SNORD64 (NR_001294)	Significant up-regulated	1.10	n.d.	n.d.
FAM21A, FAM21B, FAM21C	Significant up-regulated	1.09	n.d.	n.d.
EFEMP1 (NM_001039348)	Significant up-regulated	1.09	n.d.	n.d.
KGFLP1, KGFLP2	Significant up-regulated	1.08	n.d.	n.d.
ANKRD2 (NM_020349)	Significant up-regulated	1.07	−1.4	Non-significant
PIK3R3 (NM_003629)	Significant up-regulated	1.07	n.d.	n.d.
HS3ST3B1 (NM_006041)	Significant up-regulated	1.07	1.2	Non-significant
PTPN20A, PTPN20B (NM_001042357)	Significant up-regulated	1.07	n.d.	n.d.
FAM21A, FAM21B, FAM21C	Significant up-regulated	1.07	n.d.	n.d.
APBB2 (NM_004307)	Significant up-regulated	1.06	n.d.	n.d.
DUOX1 (NM_017434)	Significant up-regulated	1.06	n.d.	n.d.
ZNF627 (NM_145295)	Significant up-regulated	1.06	n.d.	n.d.
MCOLN2 (NM_153259)	Significant up-regulated	1.06	n.d.	n.d.
APOB (NM_000384)	Significant up-regulated	1.04	n.d.	n.d.
AJAP1 (NM_018836)	Significant up-regulated	1.04	n.d.	n.d.
BNC2 (NM_017637)	Significant up-regulated	1.03	n.d.	n.d.
LOC100652860, LOC100653093, TTC6	Significant up-regulated	1.03	n.d.	n.d.
FKTN (NM_001079802)	Significant up-regulated	1.03	n.d.	n.d.
TMEM204 (NM_024600)	Significant up-regulated	1.02	n.d.	n.d.
BMS1P1, BMS1P5 (NR_003611)	Significant up-regulated	1.00	n.d.	n.d.

106 up-regulated and 101 down-regulated genes with significantly altered expression levels (log2-fold-change >+1 and <−1 and adjusted p-value <0.05) in the mutant cell line KEB7 compared to wild-type control NEB1 keratinocytes. qRT-PCR analysis of the most promising 79 up-regulated and 79 down-regulated genes was also carried out (a fold-change >±2 is statistically significant, p < 0.05)

### Bioinformatic enrichment and clustering suggest previously undescribed pathways and functions are affected in EBS-DM

We next assigned cellular themes to the differentially expressed genes by using IBD software. Functional annotation clustering allowed us to group highly related annotation terms into enriched functional categories, as shown in Table 2. The enrichment p-values of each GO term are presented as the ordered p-value of a one-sided hypergeometric test. Significance of enrichment is indicated by a p-value of <0.05. Apart from already well-known clusters such as the regulation of epithelial cell proliferation [20], cell adhesion and response to stress [12, 13], response to retinoic acid and UV [21–23], immune response [11] or fatty acid metabolism [14], our analysis also identified previously undescribed functional classes potentially implicated in EBS-DM. The latter include genes involved in morphogenesis, and genes that regulate BMP- and canonical Wnt-signaling. In addition, two enrichment clusters associated with protein tyrosine phosphatases and symporter activities, and one further cellular component cluster linked to neurofilaments, were uncovered.

**Table 2:** Gene ontology (GO) functional annotation clustering

Cluster	GO#	Term	Annotated	Significant	Expected	p value
(1)—16 clusters						
1	GO:0050680	Negative regulation of epithelial cell proliferation	42	5	0.4881	0.0001
2	GO:0001658	Branching involved in ureteric bud morphogenesis	41	4	0.4765	0.0013
3	GO:0009954	Proximal/distal pattern formation	21	3	0.2440	0.0018
4	GO:0034341	Response to interferon-gamma	19	3	0.2208	0.0013
5	GO:0034613	Cellular protein localization	36	4	0.4183	0.0008
6	GO:0090103	Cochlea morphogenesis	20	3	0.2324	0.0015
7	GO:0032526	Response to retinoic acid	51	4	0.5927	0.0029
8	GO:0006024	Glycosaminoglycan biosynthetic process	30	3	0.3486	0.0050
9	GO:0006635	Fatty acid beta-oxidation	28	3	0.3254	0.0041
10	GO:0010811	Positive regulation of cell-substrate adhesion	29	3	0.3370	0.0045
11	GO:0030513	Positive regulation of BMP signaling pathway	31	3	0.3602	0.0055
13	GO:0048704	Embryonic skeletal system morphogenesis	38	3	0.4416	0.0097
14	GO:0090090	Negative regulation of canonical Wnt receptor signaling pathway	71	4	0.8251	0.0093
15	GO:0030326	Embryonic limb morphogenesis	39	3	0.4532	0.0104
16	GO:0009411	Response to UV	41	3	0.4765	0.0119
(2)—2 clusters						
17	GO:0004725	Protein tyrosine phosphatase activity	84	5	1.0065	0.0033
18	GO:0015293	Symporter activity	43	4	0.5152	0.0017
(3)—1 clusterr						
19	GO:0005883	Neurofilament	9	3	0.1026	0.0001

Differentially regulated genes in EBS-DM KEB7 versus wild-type NEB1 cells were assigned GO terms and classified into clusters, which were further classified as biological processes (*1*), molecular functions (*2*) and cellular components (*3*). Statistical significance of each term was calculated by a one-sided hypergeometric test; a cluster with an adjusted p-value <0.05 was considered significant. Per cluster, a minimum of 3 genes (up- or down-regulated) is significant, equating to nearly 10 % of all annotated genes per enriched term

### A complementary qRT-PCR array approach confirms differential regulation of the majority of selected candidates

Typically in microarray investigations, just a few randomly selected candidates are re-analyzed by qRT-PCR analysis for confirmation of the first hits, but this can lead to a significant number of undetected false positives. In the present study we used an array-based qRT-PCR analysis to investigate 158 of the 207 (76 %) microarray hits. To reduce the number of transcripts to be able to fit our in-house 96-well qRT-PCR array arrangement, we examined 79 randomly selected up-regulated and 79 down-regulated genes (plus controls) in three independent PCR runs (biological and technical replicates). Furthermore, we based our fold-change cut-off values on the average of seven housekeeping genes (rather than on two, as has been customary in the literature [24, 25], so at to improve the reliability of the data obtained). Out of the 158 genes analyzed, significant (p-value <0.05) differences in expression (fold change set ≥+2 for up-regulated and ≤−2 for down-regulated genes) of 121 genes (~76.58 %) was confirmed, as highlighted in Table 1. Eleven genes (~6.96 %) turned out not to be significantly modulated, and 26 genes (~16.45 %) completely failed in all independent PCR runs, probably due to poorly designed primers; this would potentially give an underestimate of the number of reproducible hits.

### Western blot analysis confirms differential expression at the protein level of 3 selected candidates

**Fig. 2:**
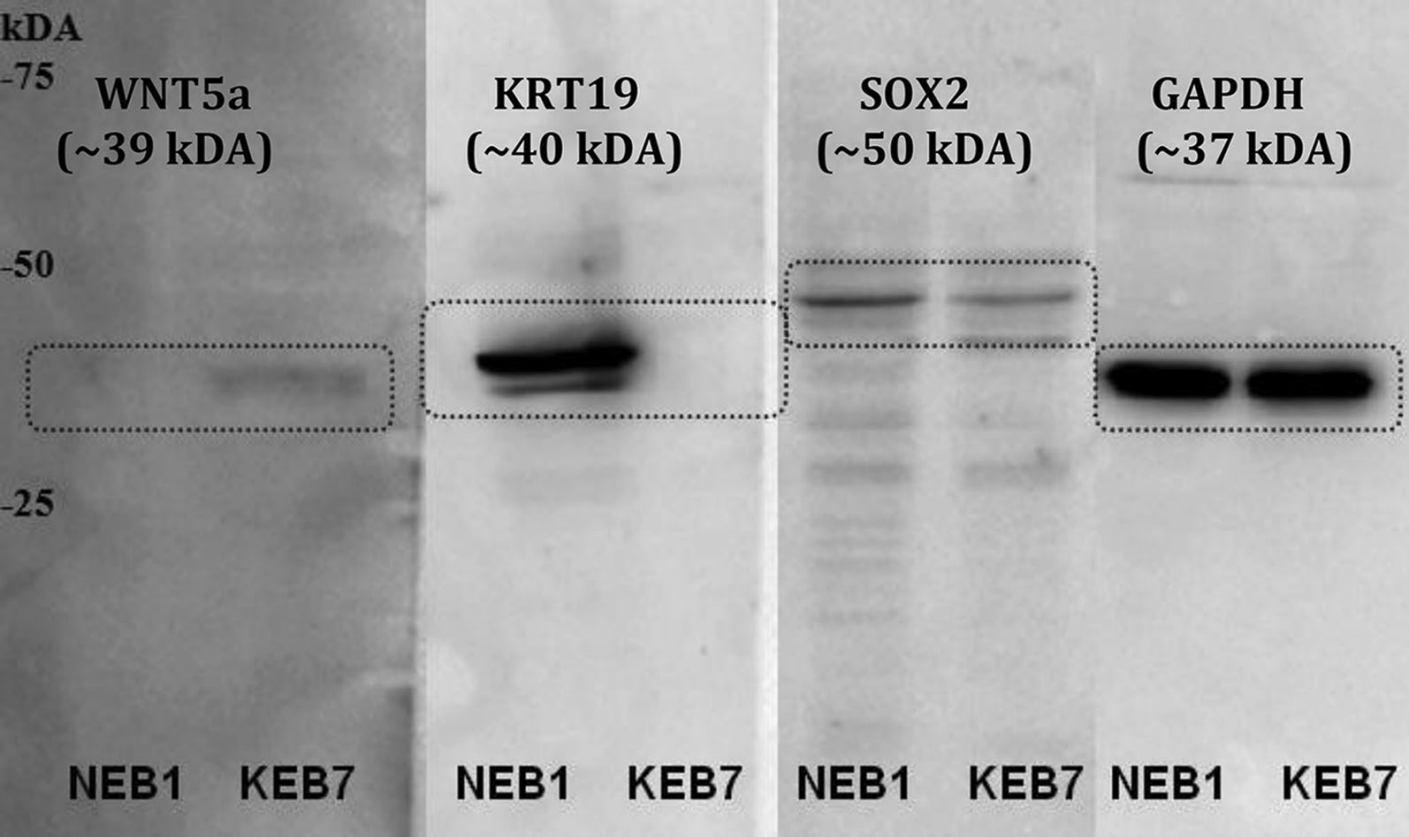
Western blot analysis for confirmation of differential expression at the protein level of 3 selected candidates—KRT19, WNT5a and SOX2. GAPDH occurred as normalization protein

Western blot analysis using crude lysates of KEB7 and NEB1 cells confirmed differential regulation of some of the selected candidates at the protein level after normalization with glyceraldehyde-3-phosphate-dehydrogenase (GAPDH), as shown in Fig. 2. Sex determining region Y-box 2 (SOX2, a log2-fold change of −1.71 by microarray analysis, no data by qRT-PCR) and cytokeratin 19 (KRT19, log2-fold change of −5.73 by microarray analysis, 1633-fold down-regulation by qRT-PCR) were both found to be significantly down-regulated in KEB7 cells, whereas wingless-type MMTV integration site family member 5a (WNT5a) was found to be significantly up-regulated (log2-fold change of 1.94 by microarray analysis, 6.9-fold up-regulation by qRT-PCR). Based on densitometric evaluation of the protein blots, SOX2 is 25-fold down-regulated, KRT19 61.7-fold down-regulated, and WNT5a 123-fold up-regulated in KEB7 cells.

## Discussion

### Global transcriptional profiling uncovers novel differentially expressed genes as well as affected pathways in the EBS-DM cell line KEB7

The present study employed global high-throughput microarray analysis together with bioinformatics-assisted functional clustering and enrichment to identify 207 differentially expressed genes in the EBS-DM cell line KEB7. A subset of 158 genes was subjected to further investigation by qRT-PCR, of which three quarters (76.58 %) could be validated as being differentially regulated. Parenthetically, the used human gene 1.0 ST Array from Affymetrix also has the ability to detect alternative mRNA isoforms. The mRNA source of the array transcripts arise from the reference sequence database (RefSeq). Each of these transcripts is read out by 26 different oligonucleotides (25mere), which are spread about the whole length of the transcription unit. Hence, a averaged probe set consists of 26 independent measure points per transcript, which are summated in only one signal. Because we have not taken into account this feature of the array to detect these alternative mRNA isoforms or splice variants we designed for qPCR validation only primer pairs for one transcript variant. Therefore, the number of our reproducible hits could potentially be underestimated.

In addition to verifying genes from previous studies (and thereby supporting their potential role in EBS pathology), a novel contribution of the present work is its uncovering of genes involved in the BMP-signaling pathway, the canonical Wnt-receptor signaling pathway, and the response to retinoic acid, suggesting that dysregulation of these processes as well may play a role in disease pathology. It is well demonstrated that keratinocyte differentiation is closely linked to hormonal action, particularly to calcitriol- [26, 27] and retinoic acid-signaling [21, 23]. In the present study, we identified four differentially regulated genes in KEB7 cells having roles in retinoic acid signaling: three genes, HSD17B2 (hydroxysteroid (17-β) dehydrogenase 2), SOX2 and MEST (mesoderm specific transcript), are down-regulated, and DKK1 (dickkopf Wnt signaling pathway inhibitor 1) is up-regulated. These potential disturbances to retinoic acid signaling may at least partially account for the observations by Wagner et al. [15] of aberrant differentiation processes in KEB7 cells. Generally, retinoids are considered to play a role in normalization of keratinocyte differentiation by down-regulating desmosomal proteins, exerting anti-proliferative effects, and regulating lipid-synthesis, growth factors, and cytokines [22, 23, 28, 29]. An interesting point in this regard is the ability of retinoic acid to act as an antagonist of Jun N-terminal kinase (JNK)-signaling, mediated by activating protein 1 (AP1) [30], since JNK/mitogen activated protein kinase (MAPK) signal transduction has previously been shown to be dysregulated in EBS-DM model keratinocytes [9, 31, 32]. Furthermore, we recently advanced a positive feedback model, suggesting that mutant KRT14 leads to an increase in AP1-dependent expression of KRT14, which in turn amplifies the level of aberrant JNK stress-signaling [33]. Concerning the canonical Wnt-receptor signaling pathway, enrichment clustering applied in our present study identified four significantly dysregulated genes: GPC3 (glypican3) and SOX2 are both down-regulated, and WNT5a and PRICKLE1 (prickle homolog 1) are up-regulated. Interestingly, WNT5a is one of the so-called non-canonical Wnt ligands. During normal development, WNT5a is secreted and directs the migration of target cells along concentration gradients. However, deregulated WNT5a signaling facilitates invasion by multiple tumor types into contiguous tissues. EB *per se* is often associated with carcinoma development, mostly in EB dystrophicans (DEB) and junctionalis (JEB) [34–36], but rare cases of carcinoma in EBS have also been described [36]. To date, the roles of WNT5a in cutaneous squamous cell carcinoma (SCC) and basal cell carcinoma (BCC) as well as the effect of WNT5a on keratinocyte migration have not been fully investigated, although Pourreyron et al. [37] recently demonstrated up-regulation of WNT5a in SCC/BCC and its localization to the leading edge of tumors as well as in tumor-associated fibroblasts. Regarding BMP signaling, enrichment clustering showed positive regulation of this pathway in EBS by highlighting three genes, GPC3, MSX2 (Msh homeobox 2) and ILK (Integrin-linked kinase), as being significantly down-regulated. BMPs are secreted signaling polypeptides belonging to the transforming growth factor-β (TGF-β) superfamily that act as important multifactor players in the development of vertebrate skin (and appendages). BMPs thereby not only regulate hair follicle (HF) morphogenesis and keratinocyte differentiation in both the HF and epidermis, but are, along with other interacting growth factor family members, also involved in normal postnatal tissue remodeling and homeostasis [38, 39]. Furthermore, BMP signaling acts as a potent tumor suppressor in the skin, inhibiting mainly epidermal- and HF-derived tumor formation [38, 40]. Consequently, dysregulation of this pathway can lead to abnormal skin development and tumor formation. Progress in this research area could be interesting to get a better knowledge of the disease pathology.

### KRT19—a novel biomarker in EBS-DM?

A further example from our list of dysregulated genes is the smallest known acidic keratin, KRT19 (significantly down-regulated in our microarray analysis, independently confirmed by both qRT-PCR and western blotting). In our previous study, in which we applied a similar whole-transcriptome microarray methodology to calcitriol-stimulated human primary keratinocytes, we [41] found a significantly increased expression level of KRT19 after treatment with the indicated secosteroid. Hence, irrespective of the exact role of KRT19 in the disease mechanism, it could constitute another useful biomarker in EBS.

## Conclusions

In summary, our study employed global high-throughput microarray analysis plus bioinformatics-assisted functional clustering and enrichment to uncover 207 differentially expressed genes in the EBS-DM cell line. Thereof, 158 genes were subjected to further investigation by a complementary qRT-PCR analysis, of which 76.58 % could be validated as being differentially regulated. We here present a pool of novel candidate genes and potential affected pathways, which may play a role in the disease pathology.

## Additional files

10.1186/s13104-015-1783-7 Literature survey (2007 to date) focusing on differential gene expression profiling in EB, especially EBS. Overview of already identified deregulated candidate genes which might play a role in the severe pathologic phenotype observed in patients with this disease.
10.1186/s13104-015-1783-7 Primer sequences of genes regulated in the EBS-DM model keratinocyte cell line KEB7 identified by microarray analysis (r = revers).

## Abbreviations

AP1: activating protein 1
BMP: bone morphogenetic protein
Ct: cycle threshold
EBS-DM: epidermolysis bullosa simplex Dowling-Meara
FDR: false discovery rate
GAPDH: glyceraldehyde-3-phosphate dehydrogenase
GO: gene ontology
HF: hair follicle
IF: intermediate filament
JEB: EB junctionalis
JNK: Jun N-terminal kinase
KRT5/14: keratin 5/14
LIMMA: linear model for microarray data
MAPK: JNK/mitogen-activated protein kinase
qRT-PCR: quantitative real-time polymerase chain reaction
RT: room temperature
SCC: cutaneous squamous cell carcinoma
SOX: sex-determining region Y-box 2
Wnt5a: wingless-type MMTV integration site family5a
MSX2: Msh homeobox 2
ILK: integrin-linked kinase
