# Supplementary material for: Whole-transcriptome gene expression profiling in an epidermolysis bullosa simplex Dowling-Meara model keratinocyte cell line uncovered novel, potential therapeutic targets and affected pathways
Source: BMC Res Notes. 2015 Dec 15;8:785. doi: 10.1186/s13104-015-1783-7 (PMC4678661; doi:10.1186/s13104-015-1783-7)
Supplement: Supplementary file 1 — 10.1186/s13104-015-1783-7 Literature survey (2007 to date) focusing on differential gene expression profiling in EB, especially EBS. Overview of already identified deregulated candidate genes which might play a role in the severe pathologic phenotype observed in patients with this disease. [file 13104_2015_1783_MOESM1_ESM.docx]

**Table S1**: **Literature survey (2007 to date) focusing on differential gene expression profiling in EB, especially EBS.** Overview of already identified deregulated candidate genes which might play a role in the severe pathologic phenotype observed in patients with this disease.

| ***Gene expression profiling studies*** | ***Model systems/cell lines*** | ***Identified genes (statistically significant deregulated in EB)*** | ***Overlap with genes identified in the present study*** | ***References*** |
| --- | --- | --- | --- | --- |
| mouse MGU74Av2 Affymetrix  expression profile of KRT5^-/-^  EBS mouse epidermis – analysis reveals a  **regulation of inflammatory cytokines** | KRT5^-/-^ EBS mouse epidermis, human HaCaT  keratinocytes stably transfected with  wt KRT14 or with KRT14 (Arg125Cys),  human MCF7-wt KRT14-, and MCF7-KRT14 Arg125Cys-transfected cells | CXCL2, CXCL1, HPRT, IL6, Keratin complex 2, basic gene 6a |  | *Lu et al. 2007* |
| expression profiles of two keratin-mutant cell lines from EBS patients compared to a control keratinocyte line before and after challenge with **hypo-osmotic shock** suggest differential regulation of **dual-specificity phosphatases** and downstream targets in EBS cells | keratinocyte cell lines KEB4 (mild EBS-loc phenotype, KRT14 mutation V270M) and  KEB7 (severe EBS-DM phenotype,  KRT14 mutation R125P)  NEB1 wt control | MKP1, MKP2, MKP3,  MKP5 and hVH3,  ERK and p38 |  | *Liovic et al. 2008* |
| microarray analysis showed down-**regulation of intercellular junction proteins** in the KEB7 cells in comparison to wild-type NEB1 cells | KEB7 and NEB1 cells | GJA1, DSG3, DP (I and II) and JUP |  | *Liovic et al. 2009* |
| gene expression profiling  (Affymetrix Genechip -  Human Gene 1.0 ST microarrays containing 28.869 oligonucleotides) revealed  **disruption of fatty acid metabolism** in epidermal tissue of EBS patients | EBS2 and EBS6 cells (severe phenotype  EBS-DM), EBS1, EBS3, EBS4 and EBS5 cells (moderate phenotype EBS-loc) | FADS1, CYP4F8, AWAT1, ALOX15B, ACSM3, SOAT1, SLC27A2, HAO2, INSIG1, KRTAP5-8, KRT25, KRT27, KRT71, KRT74, TCHH, CHI3L1, MUC1, SLCO4C1, TGIF2L, IGLJ3, IGJ, IGHA1, TMEM56 and LRRC37A2 | **TCHH** | *Bchetnia et al. 2011* |
| microarray with PCR products of 9738 ESTs of the Incyte Human UniGEM Microarray clone set showed **similarities in gene expression profiles of 3 EB subtypes** | GABEB (subtype of JEB, homozygous mutation 4003delTC in COL17A1 and PLEC), RDEB (COL7A1 mutations 7786delG and R578X) and EBS-MD (PLEC mutations 1287ins3 and Q1518X) | COL16A1, G0S2, FN1, RPS27A and LDLR |  | *Knaup et al. 2012* |
| hypothesis-generating microarray approach to identify deregulated genes in the EBS model cell line in vitro,  focusing on **cytokeratins,**  **chemokines, kallikrein-related peptidases and matrix metalloproteinases**,  additional analysis of **in vivo relevance within patient-derived blister fluids** | KEB7 cells, NEB1 cells, EBDM1 cells (skin biopsy of a five-year-old EBS DM-patient heterozygous for a KRT14 R125H mutation) | KLK1-15, MMP1-3, MMP7-17, MMP19-21, MMP23b-28*,* ACTA1, ACTA2, ACTB, ACTBL2, ACTC1, ACTR2, ACTR3, ARHGEF4, ARHGEF9, ARHGEF37, CDC42, CDC42BPA, CDC42BPB, CDC42BPG, EZR, MSN, RAC1,RDX, RHOA, WASF1, WASF2,WASF3, WAS, WIPF1,KRT1, KRT5,  KRT6A/B, KRT8, KRT10, KRT14-18, DSC1-3, DSG1-4, GJA1, GJA3-5, GJA8-10, GJB1-7, CXCL1-14, CXCL16 and CXCL17 | **KLK5**  ***GJB6***  **ARHGEF9** | *Lettner et al. 2013* |

* Abbrevations: HaCaT - [immortal](http://en.wikipedia.org/wiki/Immortalised_cell_line) human [keratinocyte](http://en.wikipedia.org/wiki/Keratinocyte) line, EBS-loc - EBS localized, wt - wild type, Arg – arginine, Cys - cysteine, MCF-7 - Michigan Cancer Foundation-7, ESTs - Expressed Sequence Tags, GABEB - generalized benign EB, JEB - junctional EB, RDEB - recessive dystrophic EB, EBS-MD - EBS with muscular dystrophy
