# Supplementary material for: Whole-transcriptome gene expression profiling in an epidermolysis bullosa simplex Dowling-Meara model keratinocyte cell line uncovered novel, potential therapeutic targets and affected pathways
Source: BMC Res Notes. 2015 Dec 15;8:785. doi: 10.1186/s13104-015-1783-7 (PMC4678661; doi:10.1186/s13104-015-1783-7)
Supplement: Supplementary file 2 — 10.1186/s13104-015-1783-7 Primer sequences of genes regulated in the EBS-DM model keratinocyte cell line KEB7 identified by microarray analysis (r = revers). [file 13104_2015_1783_MOESM2_ESM.docx]

**Table S2:** Primer sequences of genes regulated in the EBS-DM model keratinocyte cell line KEB7 identified by microarray analysis (r = revers).

| **Sequence** | **Sequence (5' - 3')** |
| --- | --- |
| **Name** |  |
| HS3ST3B1 | CCATCCAGATCGGCATCTAC |
| CAPNS2 | GAGGCCTCAGAAAGTGAGGA |
| HIST1H4A | GTGTGAAGCGGATCTCTGGT |
| ZNF570 | AGCATTTAGCCTTCGTGCAT |
| POPDC2 | CTTTTTGGCTTCCTGAGTGC |
| HOXD11 | AAAGCGCTGTCCCTATACCA |
| ZNF114 | GGCACACAACACTCATGGTC |
| SLC24A3 | GGCTTATACCAACGGGGAAT |
| CDK14 | AAGGAGTTGCTGCTTTTCCA |
| GALNT5 | TCAAGGTGTGGATGTGTGGT |
| PPP1R16B | CGGACAGGACCAACCTGTAT |
| CLEC2B | ATGCCCCTATGATTGGATTG |
| SYCP2 | ACCGAAATCAAAACCACAGC |
| SLC9A2 | CAGTGTCAGCGAAACCTTGA |
| ANKRD2 | TGTGGATGAGGAGACCTTCC |
| PRDM5 | AAAAGATCGCCTTGGCTGTA |
| SGMS1 | GAGCCTCTGGAGCATTTCAC |
| GIPC2 | CTTCGCCTGAGATCAAAAGG |
| CYYR1 | ACGCCCTACTGTTGCTCCTA |
| CSTF2T | GGATGCAAGAGGATTGGAGA |
| TSPYL5 | GGCATGATCTCCAGTCCCTA |
| PIGU | GATTTCATCCCCGCAGTCTA |
| ZNF32 | AGGGTCTATGAGTGCCAGGA |
| DSEL | GGGCAAACAGCTTCTCCATA |
| DKK1 | CCTTGGATGGGTATTCCAGA |
| MERTK | GACTTCAGCCACCCAAATGT |
| AOX1 | GTGGTGGACATCATGACAGC |
| KYNU | CCAAGAGAGGGGGAAGAAAC |
| HOXA9 | AATAACCCAGCAGCCAACTG |
| HOXC10 | AGAGCGGAAGGAAGAAGAGG |
| MSX2 | TTACCACATCCCAGCTCCTC |
| HOXD10 | GGAATGCAAACCTGTGGACT |
| ZFP42 | ACCCATCCTGGAAGAGGACT |
| H2AFY2 | TCCACTGTCACATCCCTCAG |
| RB1 | TTCACCCTTACGGATTCCTG |
| GLDC | GGCTGGAGCATATTGCTAGG |
| PTPN20A | TTCATGAGCAGACAGCCATC |
| PTPN20B | TTCATGAGCAGACAGCCATC |
| IL17RB | TTGAGGGACCTCCGAGTAGA |
| NID1 | CTTTGACTGCGTGGACAAGA |
| LMF1 | GGCGTACTACCTGCACCACT |
| CDC14B | CTGGCTGGAAGGGGACTATT |
| PPARGC1A | AGAGGCAGAAGGCAATTGAA |
| KLK5 | AAAAGGTGCGAGGATGCTTA |
| GTF2H2 | TCGTATGGGATTTCCTCAGC |
| MOXD1 | GTGCACCACATCCTGCTCTA |
| DPYD | TGTCCCTGAGGAGATGGAAC |
| C10orf99 | TAGTCCTTTCCAGCCTGCTC |
| REPS2 | GTGTGCCAGCTACCAAGTCA |
| KRT19 | GGTCAGTGTGGAGGTGGATT |
| ADHFE1 | CACCTGGAGATGGCAGAAAT |
| ARHGAP29 | CTCCAGATTAGTGGGGACCA |
| GTF2H2D | TCGTATGGGATTTCCTCAGC |
| HSD17B11 | GGTGAAGGCAGAAATTGGAG |
| ADAMTSL3 | GAACGAAGATGGCCAGAGAG |
| BCL11A | CAGCTCAAAAGAGGGCAGAC |
| IFITM3 | ATGTCGTCTGGTCCCTGTTC |
| CCDC144A | CACCTTGCGTTGGAAATCTT |
| LRCH2 | CCCTGACTCTGGAATTGGAA |
| UAP1 | CCAGACAAACCCAATGGAAT |
| PNMAL1 | AGAGACCCTACCCAGGATGC |
| NEFM | TCCTCCTATAAGCGCAGCAT |
| TDRD12 | TGGCAGAATGTTTCCTTGTG |
| FAM102B | GAAAATCGCTGAGCCAAATC |
| SGK1 | TGCTGCTGAAATAGCCAGTG |
| SMARCA1 | AAACAGAAGCTTGGCATGGT |
| AMOT | TTCTGGCTCTGGAAGCTGAT |
| IFI44L | GTTTTATGGCCACCGTCAGT |
| GLRX | GCCCAAGAGATCCTCAGTCA |
| ARHGEF9 | AGTGGCAGGCTTCTGTCCTA |
| ENPP1 | GCCTGTTCAGATGACTGCAA |
| CYP4V2 | AGCCGTCATCATTCCCTATG |
| WISP3 | CAGTTCTGCTGCAGGGTACA |
| PLA2G7 | TTGATCATGGAAAGCCAGTG |
| EYA4 | TGGCTGTAACCCTTGGACTC |
| ZFAND4 | CGAAATCTCCAGGAATGGAA |
| FAM198B | CTGGCTGAGCAAAGATGACA |
| FKBP10 | ATGGACCTCAACAAGGATGG |
| CYP7B1 | GACGAAATTGACCGTTTGCT |
| B2M | CTCACGTCATCCAGCAGAGA |
| B2M | CTCACGTCATCCAGCAGAGA |
| HPRT1 | TGCTCGAGATGTGATGAAGG |
| HPRT1 | TGCTCGAGATGTGATGAAGG |
| RPL13A | TACGCTGTGAAGGCATCAAC |
| RPL13A | TACGCTGTGAAGGCATCAAC |
| GAPDH | ACCCAGAAGACTGTGGATGG |
| GAPDH | ACCCAGAAGACTGTGGATGG |
| ACTB | GGCATCCTCACCCTGAAGTA |
| ACTB | GGCATCCTCACCCTGAAGTA |
| Anxa1 | GCAGGCCTGGTTTATTGAAA |
| Anxa1 | GCAGGCCTGGTTTATTGAAA |
| Tub | ACCTTCAGTGTGGTGCCTTC |
| Tub | ACCTTCAGTGTGGTGCCTTC |
| HS3ST3B1r | GAAGCCCTTGGTCTTGTTGA |
| CAPNS2r | ACCAGTCGTGTCACTGTCCA |
| HIST1H4Ar | TAACCGCCAAAGCCATAAAG |
| ZNF570r | GCTGAGCAAGGTAGGCAATC |
| POPDC2r | CAGGCACAGCGTCTTGTAGA |
| HOXD11r | CTGCAGACGGTCTCTGTTCA |
| ZNF114r | GGGGCATTTATGGGTTTTCT |
| SLC24A3r | CTCGTCCTCCTCTTCCTCCT |
| CDK14r | AGGTCCTCTGCATGGTTCAC |
| GALNT5r | CTTGGTCGATGAGGTGGTCT |
| PPP1R16Br | CTCGTGGGATCTTGGTAGGA |
| CLEC2Br | CCCATTGTCCTGTTCGATTT |
| SYCP2r | GGTTGCTTTTCGTGGAAGTC |
| SLC9A2r | GGCAATGATGAACTGGTCCT |
| ANKRD2r | CGATCCTGGAAGTCCACAGT |
| PRDM5r | CGCTGTGCACTGAAGAACAT |
| SGMS1r | GGGATGTCTACGCCAATGTT |
| GIPC2r | GAGTTTCGTCAAGTGCCACA |
| CYYR1r | GGAGGAGACGGTGTTGATGT |
| CSTF2Tr | CTGCATGCCTGTTCCTTGTA |
| TSPYL5r | GCCTGCCTTCTTTTTCCTTT |
| PIGUr | CATGAAGAAGATGGGGTGCT |
| ZNF32r | TGAAGCTTTTCCCACACTCC |
| DSELr | TAGCTTCCATAGGCCACACC |
| DKK1r | TCCATGAGAGCCTTTTCTCC |
| MERTKr | GGGCAATATCCACCATGAAC |
| AOX1r | AGCGGCTCCAAGTCTTGATA |
| KYNUr | CATTTCCAACTGCATGTGCT |
| HOXA9r | ATTTTCATCCTGCGGTTCTG |
| HOXC10r | GATCCGATTCTCTCGGTTCA |
| MSX2r | GCATAGGTTTTGCAGCCATT |
| HOXD10r | GCAGCAATTGGATTCTTCCT |
| ZFP42r | GGAAGCTTCTTGCCTGTCAT |
| H2AFY2r | ATCAAAGTGGGCTGAGATGG |
| RB1r | GCACTTCTTTTGAGCACACG |
| GLDCr | TACCAAGTGTGCCATCCTCA |
| PTPN20Ar | AAGAGGAACGCGTGTTGAAT |
| PTPN20Br | AAGAGGAACGCGTGTTGAAT |
| IL17RBr | TGAGTCTGGAAGGCCTCTGT |
| NID1r | TCCAGCTTCGCCACTTCTAT |
| LMF1r | GGCACCATAGTCAGCCAGTT |
| CDC14Br | AGGCCCGAAGTCTATCACCT |
| PPARGC1Ar | TTTCAAGAGCAGCAAAAGCA |
| KLK5r | TGAACTTGCAGAGGTTCGTG |
| GTF2H2r | GGGGAGCAGACACCAAAGTA |
| MOXD1r | ATGCGGATCTAATGGAGTGC |
| DPYDr | AACCAAAGGCACTGATGACC |
| C10orf99r | GCATGGTTTACAGAGCCTCA |
| REPS2r | TTTCCTACGAATGGGCTTTG |
| KRT19r | TCAGTAACCTCGGACCTGCT |
| ADHFE1r | AAGCTTGGTGACCCTTTCCT |
| ARHGAP29r | CCCCCTGATACTGATGATGG |
| GTF2H2Dr | GGGGAGCAGACACCAAAGTA |
| HSD17B11r | CCGAAGCCACAGTGACAATA |
| ADAMTSL3r | ACACTGGGGTCTCTGAATGC |
| BCL11Ar | GTAAACGTCCTTCCCCACCT |
| IFITM3r | TGACGATGAGCAGAATGGTC |
| CCDC144Ar | GAGGAGAAGGACGAGCACTG |
| LRCH2r | TGTTGGGGTCAACAAAATCA |
| UAP1r | AGGACCCAGCAATGATGAAG |
| PNMAL1r | GCAGAAGATGATCTGCACCA |
| NEFMr | CTCTATGTAGCCGGCAAAGC |
| TDRD12r | AGCTGCATTGTCCCACTTCT |
| FAM102Br | GTCCTTCTTCTTCCGCACTG |
| SGK1r | CCTCAGGTGCGAGATACTCC |
| SMARCA1r | TCCTTTTCCAGGAGCTCAAA |
| AMOTr | TGGCCATCAAGATTTCTTCC |
| IFI44Lr | TGGACTTTCCAGACCCAACT |
| GLRXr | AGCAGTTCCCCACTCTGTTG |
| ARHGEF9r | GTCAATGCGGCCTTTGTAGT |
| ENPP1r | AGAAGTCCACCCCAAGTGTG |
| CYP4V2r | TCAGGATGCACGAAAGAATG |
| WISP3r | GGTTGCTTGGCACAGATTTT |
| PLA2G7r | TTCATCACCCAGTGGAAACA |
| EYA4r | GGCAAACAAAGGTTTGCACT |
| ZFAND4r | GAGTCAGCAAGCTCCTGGTC |
| FAM198Br | GGAAGGCAAACACCTCACTC |
| FKBP10r | GCTCCTCGTCCTCATCTGAC |
| CYP7B1r | CAGTAGTCCCCGGTCTCTGA |
| B2Mr | TCTTTTTCAGTGGGGGTGAA |
| B2Mr | TCTTTTTCAGTGGGGGTGAA |
| HPRT1r | TCCCCTGTTGACTGGTCATT |
| HPRT1r | TCCCCTGTTGACTGGTCATT |
| RPL13Ar | AACACCTTGAGACGGTCCAG |
| RPL13Ar | AACACCTTGAGACGGTCCAG |
| GAPDHr | TTCTAGACGGCAGGTCAGGT |
| GAPDHr | TTCTAGACGGCAGGTCAGGT |
| ACTBr | GGGGTGTTGAAGGTCTCAAA |
| ACTBr | GGGGTGTTGAAGGTCTCAAA |
| Anxa1r | GCTGTGCATTGTTTCGCTTA |
| Anxa1r | GCTGTGCATTGTTTCGCTTA |
| Tubbr | GTGGCTGAGACAAGGTGGTT |
| Tubbr | GTGGCTGAGACAAGGTGGTT |
| SLC44A5 | GACTTGTGGCCTGGGTACAT |
| GJB6 | GCAGCATCTTTTTCCGAATC |
| NAP1L5 | CCCTCAAAAAGCTGCAGAAG |
| NEFH | ACTCCCCAAAATTCCCTCTG |
| ZNF502 | TGCAGTGAATGTGGCTCTTC |
| SLC16A9 | GGAGGGTTTCCACCTTCATT |
| AHI1 | CCGCTCTATGATGGCTCTTC |
| TP53INP1 | GGGGCAGCATATTCATTGTT |
| HSD17B2 | GGGAAAAGCTGGAGAAGGAC |
| IFFO2 | CTCTGGGAGGATTTCACCAA |
| GABPB2 | AAAGCAAGGAAGGCAATGAA |
| FAM25C | GTGGAGGAAGTGGTGAAGGA |
| NREP | TCTGGGTCAGTCAAGAACCA |
| DENND1B | AGAGCACAGGCTGCTTTGTT |
| ACOXL | TTGGGTTGACGATAGCCATT |
| SLC38A4 | GGCTTCTTCTGCCACTATGC |
| SLC2A3 | AAGGGCAGGAAGAAGGACTC |
| ASAH2 | ACGGGCCATGTATCAGAGAG |
| PRICKLE1 | TGAACTCTTCCATGCTGCAC |
| DDX43 | GGAGATCGGCCATTGATAGA |
| AKR1B10 | ATCACCGTTACGGCCTACAG |
| RPL10 | CGTACCCAAAGTCTCGCTTC |
| TMPRSS15 | ATGCAAGGCAGACCATTTTC |
| ROBO1 | GGTGTCTGGGCAATGAAACT |
| SLC15A2 | TGACAGTGGTGGGAAATGAA |
| STOX1 | CCAGACTGGACCAAAGGAAA |
| AHCY | CGTCATCATCACCGAGATTG |
| GPC3 | CTGGATGAGGAAGGGTTTGA |
| SLC7A2 | CCTAGCTTTCCTCGTGTTGG |
| EVC2 | GGTCGAGTGAAGCACAGACA |
| SMOC2 | CAGCCGAAATGTGACAACAC |
| KIAA1324L | CCACGGGTTCTGAACTAGGA |
| WDR17 | TCCGTATACACCCCCTTCTG |
| BGN | GCTTCGCTACTCCAAGCTGT |
| GPR143 | CCACCGTGTGGTTAGGATTC |
| STRBP | GTGAGCGAGACCCAACAGAT |
| ELAVL2 | CGCCCAAGTTCAGCTTCTAT |
| LRP12 | GAATGCTGCAAGGGGATTTA |
| SATB2 | CTTTGCAAGAGTGGCATTCA |
| NLRP2 | ATGCTAGACTGGGCAGAGGA |
| MSLN | CAAGAAGTGGGAGCTGGAAG |
| PCCA | TCCGTGTTGCTAAGGGCTAC |
| ZNF136 | TGTGGGAAACCATGAGGAAT |
| TMTC1 | TTCCTACCTCTTGGCCTTCA |
| ARHGAP28 | GTGATGGCACCAAACCTTTT |
| RNF212 | CCTGCGAGAATCTCCATGAT |
| LY75 | CAAGTGCATCAAGCCAGTGT |
| CPNE1 | CAAGCCCTGAGTTCTCCAAG |
| CPT1C | CAGTGACTGGTGGGAGGAAT |
| BLMH | GTGGTTTGGCTGTGATGTTG |
| PDZK1 | TGGGCTAGAGGATGAGGATG |
| SLC16A4 | CTGGTAGCCAGAGCCAAAAC |
| OLFM4 | AGCTCTTTCCCAGGTGTTGA |
| PITRM1 | TCACCGTAAACCAGGTGACA |
| GHR | GTGGCTCCTCACATCAAGGT |
| MEST | CGCAGGATCAACCTTCTTTC |
| WNT5A | TGGCTTTGGCCATATTTTTC |
| SLCO1B3 | GGATGCAAATCCTCAAGTGG |
| ZNF43 | GTGGCAAAGCCTTTAACTGG |
| IKZF3 | ATCAACAAGGAAGGGGAGGT |
| SELENBP1 | ATCTGGCCACTGTGGATGTT |
| PAX6 | ATGAGGCTCAAATGCGACTT |
| EPSTI1 | ACTGACCTCGAAAAGCCTCA |
| ROR1 | ACCTCGACACCACAGACACA |
| MAN1A1 | GCAGTGGAACTTGGGGTAAA |
| ZNF334 | TGCAGTGAATGCAGGAAAAC |
| CRIP2 | GCCTCCAGTGTCACCACTTT |
| TFPI2 | GGGCCCTACTTCTCCGTTAC |
| TBX18 | ACCCTCAACCGATACAGCAC |
| ITGBL1 | GAAGACCTCGATGGTGTGGT |
| ZNF700 | GCATCAGAGGTGACACTGGA |
| TCHH | AAGAGAAGAGACGCCGTCAA |
| ERCC6-PGBD3 | CAAGGAGACCTTGGGAGTCA |
| MAPK8 | AATGGTTTGCCACAAAATCC |
| IRX4 | CCAGACTGAGTTTCCGTCGT |
| EDIL3 | TCGAAGACATTGCACTTTGC |
| ARMCX2 | ATACACCAGGGGGAGAGACC |
| BST2 | TCTCCTGCAACAAGAGCTGA |
| SLC6A14 | CCTTGGTCTCGTCTGTGTGA |
| B2M | CTCACGTCATCCAGCAGAGA |
| B2M | CTCACGTCATCCAGCAGAGA |
| HPRT1 | TGCTCGAGATGTGATGAAGG |
| HPRT1 | TGCTCGAGATGTGATGAAGG |
| RPL13A | TACGCTGTGAAGGCATCAAC |
| RPL13A | TACGCTGTGAAGGCATCAAC |
| GAPDH | ACCCAGAAGACTGTGGATGG |
| GAPDH | ACCCAGAAGACTGTGGATGG |
| ACTB | GGCATCCTCACCCTGAAGTA |
| ACTB | GGCATCCTCACCCTGAAGTA |
| Anxa1 | GCAGGCCTGGTTTATTGAAA |
| Anxa1 | GCAGGCCTGGTTTATTGAAA |
| Tub | ACCTTCAGTGTGGTGCCTTC |
| Tub | ACCTTCAGTGTGGTGCCTTC |
| SLC44A5r | GGGCACTTGGAGACACAGAT |
| GJB6r | AAGCAGCATGCAAATCACAG |
| NAP1L5r | CCTCGTCATCCTCGTACTCC |
| NEFHr | CTTCTTCACCCCCTTCTTCC |
| ZNF502r | GCTCTGACAAAAGGCTTTGC |
| SLC16A9r | GGCTAGGCCCATGATGATTA |
| AHI1r | CATCCTCGTTCTCCTGCATT |
| TP53INP1r | ATGAACAACCCAGCCATTGT |
| HSD17B2r | TTTCCCTGGCGTGTAATAGG |
| IFFO2r | ATGTACTCGTGCAGGTGTCG |
| GABPB2r | GCATCTACCTCAGCCACCTC |
| FAM25Cr | GGTGATGGCATTTGTGACTG |
| NREPr | ATTCTTGGGGAGCGGAGTT |
| DENND1Br | AGAAACCCCTTCCTGCATTT |
| ACOXLr | AGCTCCTTTCCCTGGAAGAC |
| SLC38A4r | GATGCTTGGAATTTGGAGGA |
| SLC2A3r | CTGAAGAGTTCGGCCACAAT |
| ASAH2r | GGCCTCCAACTCCATCAATA |
| PRICKLE1r | TGCCGGATTTCAATGTCATA |
| DDX43r | TCCCCATCCTTCAAGTCATC |
| AKR1B10r | CGTGCTGGTGTCACAGACTT |
| RPL10r | GCCATCTTTGCCACAACTTT |
| TMPRSS15r | GTGGTCCAGTTCTCAGCACA |
| ROBO1r | CCTCAGGTGACACAGGGTTT |
| SLC15A2r | TCCCATCTTCACGAATGACA |
| STOX1r | TGCTCTGTTGAGGGTGACTG |
| AHCYr | CCACGTCAAAGTGTCCAATG |
| GPC3r | CGAGGTTGTGAAAGGTGCTT |
| SLC7A2r | ACGCTGGCAAAAATGGTAAG |
| EVC2r | AGCTGACAGCAAAGGCATCT |
| SMOC2r | GTTCTGAGAGCCTGCCTGAC |
| KIAA1324Lr | GTTCTCTGGAATGCCCATGT |
| WDR17r | CCCGTCTTCAAGGAAAAACA |
| BGNr | CACCCACTTTGGTGATGTTG |
| GPR143r | TACAGCAGGATGGTGCTCAG |
| STRBPr | TGAAGACCCAGCACCTTCTT |
| ELAVL2r | GCTTCTTCTGCCTCAATTCG |
| LRP12r | ACAGACACCATTTCGGGAAC |
| SATB2r | ACCATGCTCACATTGGGATT |
| NLRP2r | AGCTCATCAAAGCCGTCAAT |
| MSLNr | GAATGTCCTCAGGGCTCATC |
| PCCAr | ATCACTTCCTGGTTGGATGC |
| ZNF136r | CACTCCAGGGATTTTCTTGC |
| TMTC1r | CCTCCTTGTGCTCCAGTCTC |
| ARHGAP28r | CACTTGGGAGCTGCTTCTTC |
| RNF212r | ACTGAACGCTAGGAGGAGCA |
| LY75r | TCACATTTCCACCACAGCAT |
| CPNE1r | GTTTTCCAGGCTTCAGCATC |
| CPT1Cr | CTTCTCGTACTGGGCAGAGC |
| BLMHr | AAGCACCATCCTGATCATCC |
| PDZK1r | AGGGGTGTCAAGTGGATCAG |
| SLC16A4r | GTGGTGGCTAAAGGAGCAAG |
| OLFM4r | AAGCGTTCCACTCTGTCCAC |
| PITRM1r | GAGACCCACAAAGGACGGTA |
| GHRr | CAAGGCAGTCGCATTGAGTA |
| MESTr | CATCAGTCGTGTGAGGATGG |
| WNT5Ar | CCGATGTACTGCATGTGGTC |
| SLCO1B3r | TGTGGTACCTCCTGTTGCAG |
| ZNF43r | TAAGGTTTGAGGACCGGCTA |
| IKZF3r | CAGGGCTCTGTGTTCTCCTC |
| SELENBP1r | CACGTCCACCACATAGATGC |
| PAX6r | CATTTGGCCCTTCGATTAGA |
| EPSTI1r | AGCATTTCCCTGGCAGTAGA |
| ROR1r | GTGCGGTTGCCAATAAATCT |
| MAN1A1r | TTCAGCAAAGATGGGGTTTC |
| ZNF334r | TTCTTGCTGAAGGCATTTCC |
| CRIP2r | ATTCCATAGCAGGGCTTGTG |
| TFPI2r | CTTTTCTGTGGACCCCTCAC |
| TBX18r | GACATTCCCGAAATCTGCAT |
| ITGBL1r | ATGCACTTCCCACAATGACA |
| ZNF700r | TCGAATGCTTGAATGGAAAA |
| TCHHr | GGAATTTTCTCTCCCGTTCC |
| ERCC6-PGBD3r | GCACTTTCTTCTGCCGTTTC |
| MAPK8r | AGTCAGCTGGGAAAAGGACA |
| IRX4r | AAAGTGCTGTGCCTGAGGAT |
| EDIL3r | AGGTCCAGGCATTCACTTTG |
| ARMCX2r | CCAACTGTGTCCAGAGCAGA |
| BST2r | CTTCTCAGTCGCTCCACCTC |
| SLC6A14r | AGCTCTCCACCATAGCCAGA |
| B2Mr | TCTTTTTCAGTGGGGGTGAA |
| B2Mr | TCTTTTTCAGTGGGGGTGAA |
| HPRT1r | TCCCCTGTTGACTGGTCATT |
| HPRT1r | TCCCCTGTTGACTGGTCATT |
| RPL13Ar | AACACCTTGAGACGGTCCAG |
| RPL13Ar | AACACCTTGAGACGGTCCAG |
| GAPDHr | TTCTAGACGGCAGGTCAGGT |
| GAPDHr | TTCTAGACGGCAGGTCAGGT |
| ACTBr | GGGGTGTTGAAGGTCTCAAA |
| ACTBr | GGGGTGTTGAAGGTCTCAAA |
| Anxa1r | GCTGTGCATTGTTTCGCTTA |
| Anxa1r | GCTGTGCATTGTTTCGCTTA |
| Tubbr | GTGGCTGAGACAAGGTGGTT |
| Tubbr | GTGGCTGAGACAAGGTGGTT |
